# A dataset of temperature and salinity in the South Brazil Bight: Identifying water mass interfaces

**DOI:** 10.1016/j.dib.2018.08.166

**Published:** 2018-09-05

**Authors:** Marcelo Dottori, Iago Nicolai Beraldo Dalsenter, Dante Campagnoli Napolitano, Ilson Carlos Almeida da Silveira

**Affiliations:** Oceanographic Institute of the University of Sao Paulo, Brazil

## Abstract

Temperature and salinity data were recorded at the slope and outer shelf of the South Brazil Bight, from November 2014 to February 2015 using an Underwater Autonomous Vehicle, specifically, a glider. During the mission, the slope was crossed 10 times, reaching depths of up to 970 m and with a sampling rate of one record per second. The density profiles were also calculated to estimate interface levels between Tropical Water (TW) and South Atlantic Central Water (SACW), and between SACW and Antarctic Intermediate Water (AAIW). The interface levels are presented in cross section figures and the raw dataset is provided in netCDF files, including scientific and navigation datasets.

**Specifications table**

**Table:** 

Subject area	*Marine Science*
More specific subject area	*Oceanography; Physical Oceanography*
Type of data	*Figures; netCDF files*
How data was acquired	*Temperature and salinity acquired with a pumped CTD from Sea Bird on board an underwater glider model Slocum G2 (Teledyne Webb Research)*
Data format	*Figures: interpolated data; netCDF files: raw data*
Experimental factors	*From November 2014 to February 2015 salinity and temperature data were recorded using an underwater autonomous vehicle at a sampling rate of 1 record per second.*
Experimental features	*Identification of the water masses (Tropical Water, South Atlantic Central Water and Antarctic Intermediate Water) and their interfaces in the slope and outer shelf.*
Data source location	*South Brazil Bight, in the South Atlantic. Latitude and longitude are provided along with the dataset.*
Data accessibility	*Resource link:*http://www3.io.usp.br:32080/lhico/data/
Related research article	–

**Value of the data**

• Data can be used to compare and calibrate global numerical models in a region of scarce data.
• Data can be used to study the flow and meanders of the Brazil Current, as well as the salt and heat transport in the region.
• Data can be used to compare satellite measurements, including incipient satellite data of surface salinity.
• Data can be used to study thermocline, halocline and pycnocline variability.

## 1 Data

Vertical profiles of *in-situ* temperature, in °C, and salinity in the South Brazil Bight, from the surface up to 1000 m depth, are provided in netcdf files. Along with the hydrographical data, these files also contain the pressure, in dbar, the time, in days, starting in January 1st 2014, and the *σ*_0_ density, in kg m^−3^. Equivalent netcdf files also provided the location in terms of latitude and longitude, and the time of each location, in days, also starting in January 1st 2014.

This dataset comprises a total of 10 hydrographical (temperature and salinity) and 10 related location files associated with the transects performed during the mission.

**Fig. 1:**
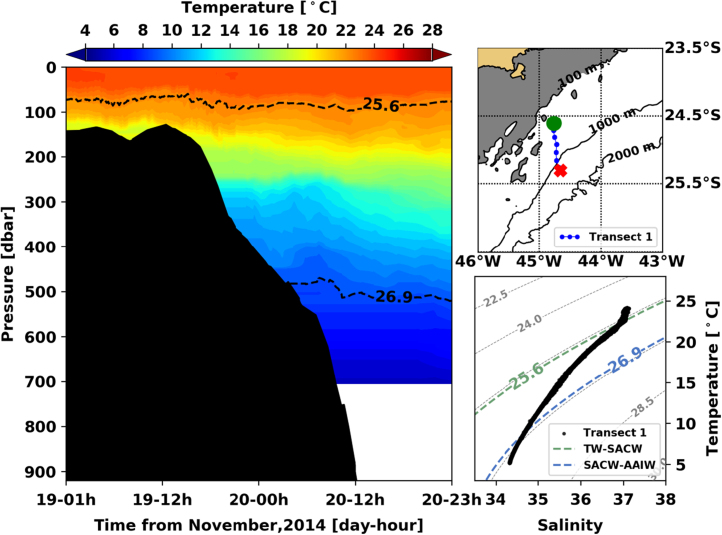
Left panel shows the interpolated temperature of transect 1. The isopycnal lines (25.8 *σ*_0_ and 26.9 *σ*_0_) show the interface between Tropical Water and South Atlantic Central Water and between South Atlantic Central Water and Antarctic Intermediate Water, respectively. Right panels show the glider trajectory (top) and the TS diagram (bottom) for the same transect. This leg of the mission took place from November 19th to 20th of 2014. The green circle indicates the beginning of the transect and the red cross, its end.

**Fig. 2:**
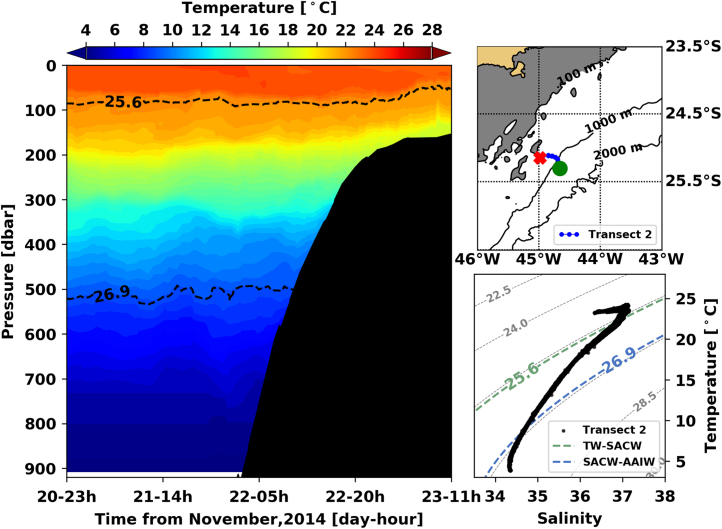
Left panel shows the interpolated temperature of transect 1. The isopycnal lines (25.8 *σ*_0_ and 26.9 *σ*_0_) show the interface between Tropical Water and South Atlantic Central Water and between South Atlantic Central Water and Antarctic Intermediate Water, respectively. Right panels show the glider trajectory (top) and the TS diagram (bottom) for the same transect. This leg of the mission took place from November 20th to 23rd of 2014. The green circle indicates the beginning of the transect and the red cross, its end.

**Fig. 3:**
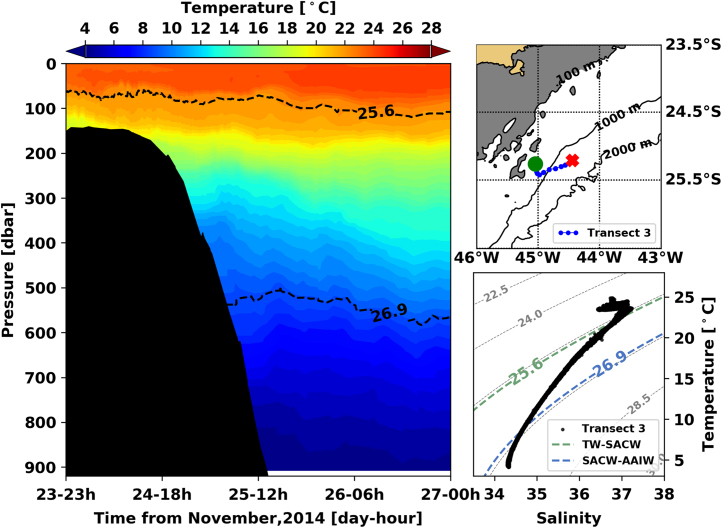
Left panel shows the interpolated temperature of transect 1. The isopycnal lines (25.8 *σ*_0_ and 26.9 *σ*_0_) show the interface between Tropical Water and South Atlantic Central Water and between South Atlantic Central Water and Antarctic Intermediate Water, respectively. Right panels show the glider trajectory (top) and the TS diagram (bottom) for the same transect. This leg of the mission took place from November 23rd to 27th of 2014. The green circle indicates the beginning of the transect and the red cross, its end.

**Fig. 4:**
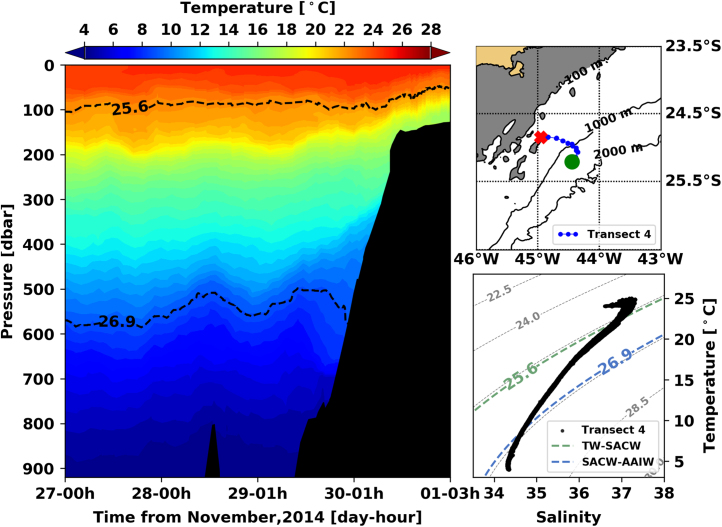
Left panel shows the interpolated temperature of transect 1. The isopycnal lines (25.8 *σ*_0_ and 26.9 *σ*_0_) show the interface between Tropical Water and South Atlantic Central Water and between South Atlantic Central Water and Antarctic Intermediate Water, respectively. Right panels show the glider trajectory (top) and the TS diagram (bottom) for the same transect. This leg of the mission took place from November 27th to December 1st of 2014. The green circle indicates the beginning of the transect and the red cross, its end.

**Fig. 5:**
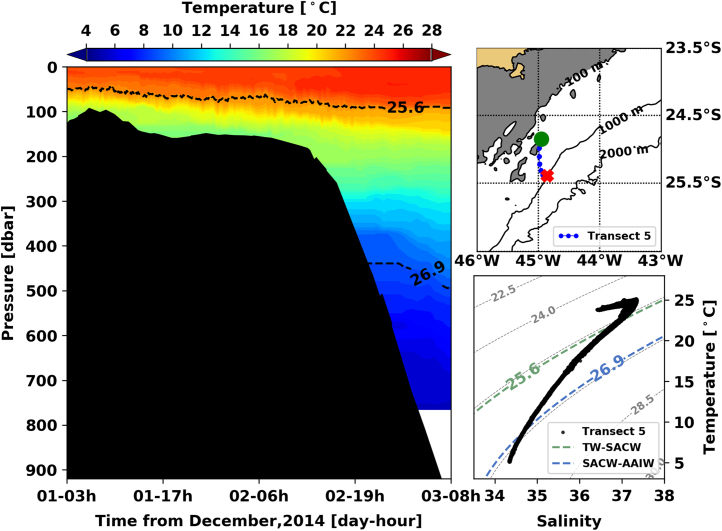
Left panel shows the interpolated temperature of transect 1. The isopycnal lines (25.8 *σ*_0_ and 26.9 *σ*_0_) show the interface between Tropical Water and South Atlantic Central Water and between South Atlantic Central Water and Antarctic Intermediate Water, respectively. Right panels show the glider trajectory (top) and the TS diagram (bottom) for the same transect. This leg of the mission took place from December 1st to 3rd of 2014. The green circle indicates the beginning of the transect and the red cross, its end.

**Fig. 6:**
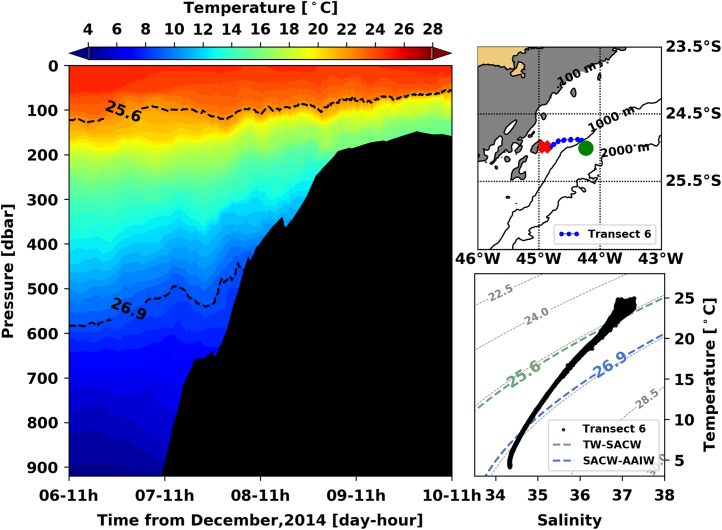
Left panel shows the interpolated temperature of transect 1. The isopycnal lines (25.8 *σ*_0_ and 26.9 *σ*_0_) show the interface between Tropical Water and South Atlantic Central Water and between South Atlantic Central Water and Antarctic Intermediate Water, respectively. Right panels show the glider trajectory (top) and the TS diagram (bottom) for the same transect. This leg of the mission took place from December 6th to 10th of 2014. The green circle indicates the beginning of the transect and the red cross, its end.

**Fig. 7:**
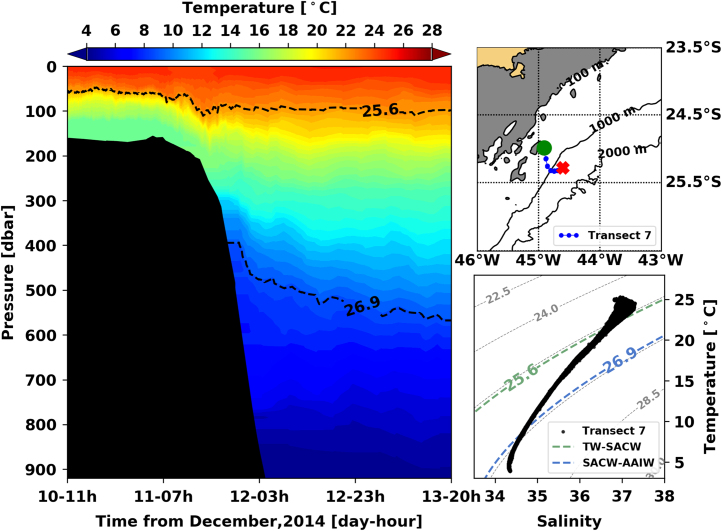
Left panel shows the interpolated temperature of transect 1. The isopycnal lines (25.8 *σ*_0_ and 26.9 *σ*_0_) show the interface between Tropical Water and South Atlantic Central Water and between South Atlantic Central Water and Antarctic Intermediate Water, respectively. Right panels show the glider trajectory (top) and the TS diagram (bottom) for the same transect. This leg of the mission took place from December 10th to 13th of 2014. The green circle indicates the beginning of the transect and the red cross, its end.

**Fig. 8:**
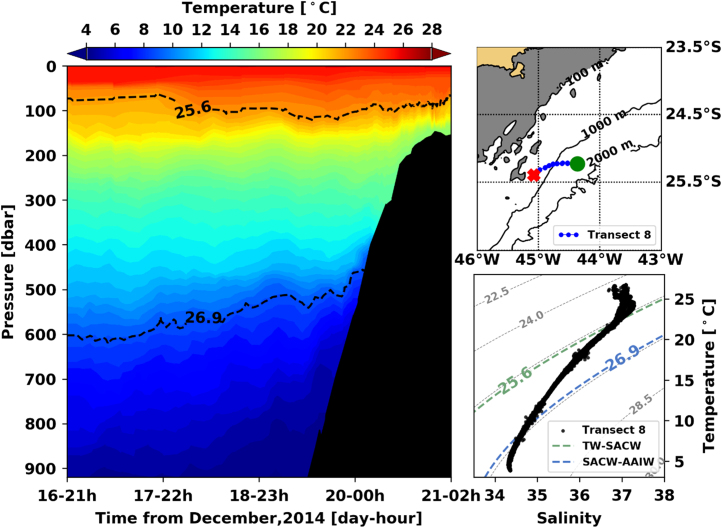
Left panel shows the interpolated temperature of transect 1. The isopycnal lines (25.8 *σ*_0_ and 26.9 *σ*_0_) show the interface between Tropical Water and South Atlantic Central Water and between South Atlantic Central Water and Antarctic Intermediate Water, respectively. Right panels show the glider trajectory (top) and the TS diagram (bottom) for the same transect. This leg of the mission took place from December 16th to 21st of 2014. The green circle indicates the beginning of the transect and the red cross, its end.

**Fig. 9:**
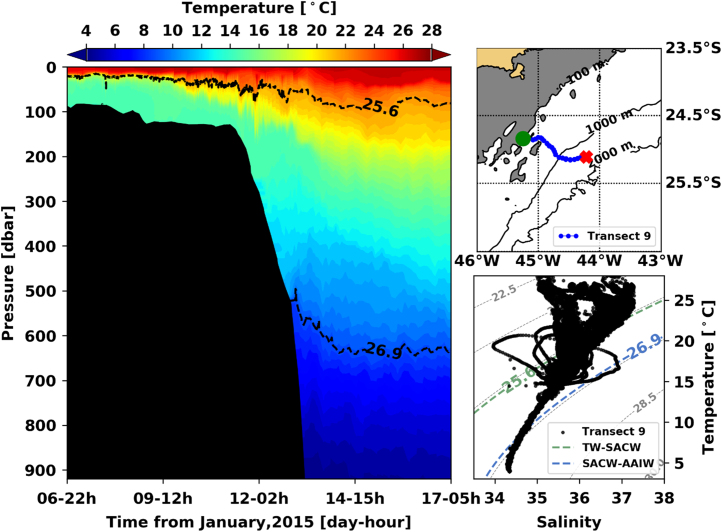
Left panel shows the interpolated temperature of transect 1. The isopycnal lines (25.8 *σ*_0_ and 26.9 *σ*_0_) show the interface between Tropical Water and South Atlantic Central Water and between South Atlantic Central Water and Antarctic Intermediate Water, respectively. Right panels show the glider trajectory (top) and the TS diagram (bottom) for the same transect. This leg of the mission took place from January 6th to 17th of 2014. The green circle indicates the beginning of the transect and the red cross, its end.

**Fig. 10:**
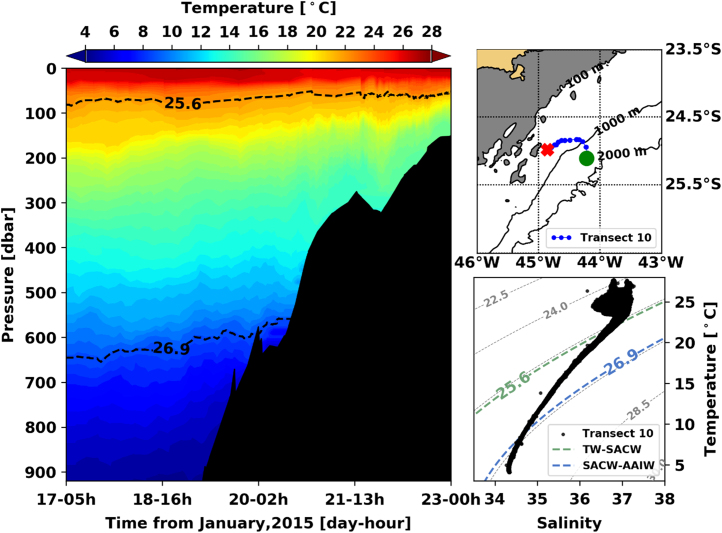
Left panel shows the interpolated temperature of transect 1. The isopycnal lines (25.8 *σ*_0_ and 26.9 *σ*_0_) show the interface between Tropical Water and South Atlantic Central Water and between South Atlantic Central Water and Antarctic Intermediate Water, respectively. Right panels show the glider trajectory (top) and the TS diagram (bottom) for the same transect. This leg of the mission took place from January 17th to 23rd of 2014. The green circle indicates the beginning of the transect and the red cross, its end.

Fig. 1, Fig. 2, Fig. 3, Fig. 4, Fig. 5, Fig. 6, Fig. 7, Fig. 8, Fig. 9, Fig. 10 present the interpolated temperature, the TS diagram and the trajectory navigated by the glider on each transect. Also, on each temperature pannel, the interface between the Tropical Water (TW) and the South Atlantic Central Water (SACW), represented by the *σ*_0_ isopycnal line of 25.6 kg m^−3^, and the interface between the SACW and the Antarctic Intermediate Water (AAIW), represented by the *σ*_0_ isopycnal line of 26.9 kg m^−^^3^, are shown.

The lowest values of temperature are found at the deepest regions sampled, at about 1000 m depth, and are slightly below 4 °C, which is compatible with the Antarctic Intermediate Water (AAIW). The highest values of temperature were recorded at the surface and are slightly above 28 °C, associated with the Tropical Water (TW). The lowest values of salinity are above 34, also associated with the AAIW at the deepest regions sampled. The highest values of salinity were recorded near the surface, around 37, and are associated with the TW.

## 2 Experimental design, materials, and methods

A glider, which is an Autonomous Underwater Vehicle (AUV), was used to sample the South Brazil Bight (SBB), crossing the slope and the outer shelf repeatedly, from mid November 2014 until late January 2015. A more detailed description of the glider functioning can be obtained in Davis et al. [1]. The glider was equipped with a SeaBird pumped CTD, model GPCTD, that collected temperature and salinity data at a sampling rate of 1 Hz. These hydrographical data were obtained in a diagonal vertical motion, following the navigation procedure of the glider. During the deployment of the AUV, vertical profiles of temperature and salinity were collected using a ship-based CTD to compare them with the data obtained with the glider CTD at the same location. Both profiles presented equivalent results, with temperature and salinity differences smaller than 0.01 °C and 0.01, respectively, and pressure differences within 0.1% of the local pressure. The same procedure was performed during the recovery of the glider, at the last day of the mission and, again, both the ship-based and the glider CTDs profiles presented equivalent results.

The *σ*_0_ density values were calculated for each pair of temperature and salinity using the International Thermodynamic Equation of Sea Water (TEOS–10) [2], [3]. Afterwards, the interface between the TW and SACW was estimated by locating the depth of the 25.8 *σ*_0_ isopycnal [4] through interpolation of the density values over depth using cubic splines. Similarly, the interface between the SACW and the AAIW was estimated by computing the depth of the 26.9 *σ*_0_ isopycnal [5].

The temperature structure presented in Fig. 1, Fig. 2, Fig. 3, Fig. 4, Fig. 5, Fig. 6, Fig. 7, Fig. 8, Fig. 9, Fig. 10 was obtained also by interpolation using cubic splines. Initially the temperature values were interpolated horizontally, using samples at the same depth. Then, the interpolation was performed vertically.
